# The limits of genetic screening: trisomy 21 after euploid preimplantation genetic testing for aneuploidy and negative noninvasive prenatal testing

**DOI:** 10.1016/j.xfre.2026.06.010

**Published:** 2026-07-06

**Authors:** Jennifer Law, Kyra Ramsey, Nicole Sprawka, Joana Perdigao

**Affiliations:** Department of Obstetrics and Gynecology, Loyola University Medical Center, Maywood, IL

**Keywords:** Preimplantation genetic testing for aneuploidy (PGT-A), noninvasive prenatal test (NIPT), cell-free DNA (cfDNA), aneuploidy, mosaicism

## Abstract

**Objective:**

To describe a case of false-negative preimplantation genetic testing for aneuploidy (PGT-A) and noninvasive prenatal test (NIPT) resulting in a neonate born with Down Syndrome.

**Design:**

Case report

**Subjects:**

A 40-year-old multiparous woman with in vitro fertilization pregnancy due to a history of tubal ligation presenting to an academic medical center.

**Exposure:**

Prenatal aneuploidy screening with PGT-A and cell-free DNA.

**Main Outcome Measures:**

PGT-A test was conducted on a day 5 blastocyst which resulted as euploid. Subsequent NIPT test was obtained at 10 weeks and reported low-risk results.

**Results:**

The patient underwent vacuum-assisted vaginal delivery of a liveborn male infant with features of Down Syndrome. Postnatal karyotype confirmed nonmosaic trisomy 21.

**Conclusion:**

PGT-A and NIPT are powerful tools used for risk reduction, but they do not eliminate risk.

Preimplantation genetic testing for aneuploidy (PGT-A) and noninvasive prenatal testing (NIPT) represent two of the most advanced tools available for aneuploidy risk reduction in assisted reproductive technology (ART) pregnancies. PGT-A was first used in 1990 by Handyside et al. (1) in the United Kingdom to determine the sex of embryos from patients with X-linked disorders. Earlier testing techniques included fluorescent in-situ hybridization; however, next-generation sequencing (NGS) with or without SNP analysis has become standard of care for blastocyst stage embryo biopsy testing (1). Blastocyst biopsy involves sampling 5 to 10 cells of the trophectoderm layer, which eventually gives rise to the placenta (2). This test screens for aneuploidy in all chromosomes, including the 22 pairs of autosomes and the sex chromosomes. However, there are inherent technical and biological limitations to PGT-A. In the case of mosaicism, for instance, PGT-A may not reflect the true genetic makeup of the inner cell mass, which gives rise to the fetus.

Both the American College of Obstetricians and Gynecologists (ACOG) and the American Society for Reproductive Medicine (ASRM) state that a negative PGT-A does not guarantee a chromosomally normal newborn. ACOG recommends that standard prenatal screening should be offered to all patients following PGT-A (3). NIPT is a maternal blood test that measures the placental cell-free DNA (cfDNA) that is circulating within maternal serum (4). The Society for Maternal Fetal Medicine recommends that cfDNA screening for common aneuploidies, including trisomies 21, 18, and 13, be made routinely available to all obstetrical patients (4). The detection rate for trisomy 21, or Down syndrome, is 99%, with a false-positive rate of 0.02% and a false-negative rate of 0.02% to 0.26% (4, 5). Some sources of error that can result in a false-positive or false-negative screening results include technical errors, such as sampling errors, DNA amplification artifacts, and varying laboratory methods. Other biological sources of error include mosaicism (in the mother, placenta, or fetus), maternal malignancy, or a demized twin (4). With the increased rates of IVF and PGT-A, clinicians must understand the screening technologies and their benefits and limitations. They should be ready to counsel on the possibility of both false-positive and false-negative results and the implications of each.

Here we report such a case of false-negative results in both PGT-A and NIPT in the same pregnancy. To our knowledge, this is one of the first reported cases in the literature of a euploid blastocyst on next-generation-based PGT-A and low-risk NIPT with adequate fetal fraction, resulting in a live-born infant with trisomy 21. This case highlights the critical importance of appropriate counseling and is a reminder that these technologies represent a risk reduction strategy, not risk elimination.

## Case report

The patient is a 40-year-old G4P1112 at 37 weeks and 5 days gestation who presents with an in vitro fertilization (IVF)-conceived pregnancy. Her medical history was notable for advanced maternal age, gestational diabetes, anxiety and cervical dysplasia. Surgical history included a prior laparoscopic tubal ligation and cold knife cone biopsy. Due to her tubal ligation, she underwent IVF at a private fertility clinic. The patient elected for PGT-A and underwent transfer of a day 5 blastocyst that was classified as euploid, 46,XY. Biopsied material was analyzed with the Embgenix PGT-A Kit (Takara Bio, USA) and run on the Illumina NextSeq NGS platform. The performing PGT-A laboratory reports an embryo as “euploid” when the estimated mosaicism is <30%. Three additional embryos were retrieved and biopsied at the same time as the transferred embryo with the following PGT-A results: 46,XY; 45,XX-15; and 45,XY,-22. At 10 weeks and 4 days, she underwent NIPT that returned with low-risk results with a fetal fraction of 10%.

At 19 weeks and 3 days, the patient had a detailed anatomy ultrasound that was overall reassuring. No fetal anomalies or soft markers were identified. The left ventricular outflow tract and aortic arch had suboptimal views; a subsequent fetal echocardiogram was performed by pediatric cardiology at 23 weeks that was normal. She developed gestational diabetes during the pregnancy, which was managed with insulin. Polyhydramnios was first noted at 32 weeks, and weekly biophysical profiles (BPPs) were performed to monitor. At the 36-week BPP, severe polyhydramnios was noted with an amniotic fluid index of 35.8 cm, as well as a double bubble sign concerning for duodenal atresia (Figure 1). These findings prompted referral back to maternal fetal medicine (MFM), where the ultrasound findings were confirmed. The association of duodenal atresia and trisomy 21 was reviewed, and an amniocentesis was offered. The patient declined with plans for postnatal testing.

**Figure 1:**
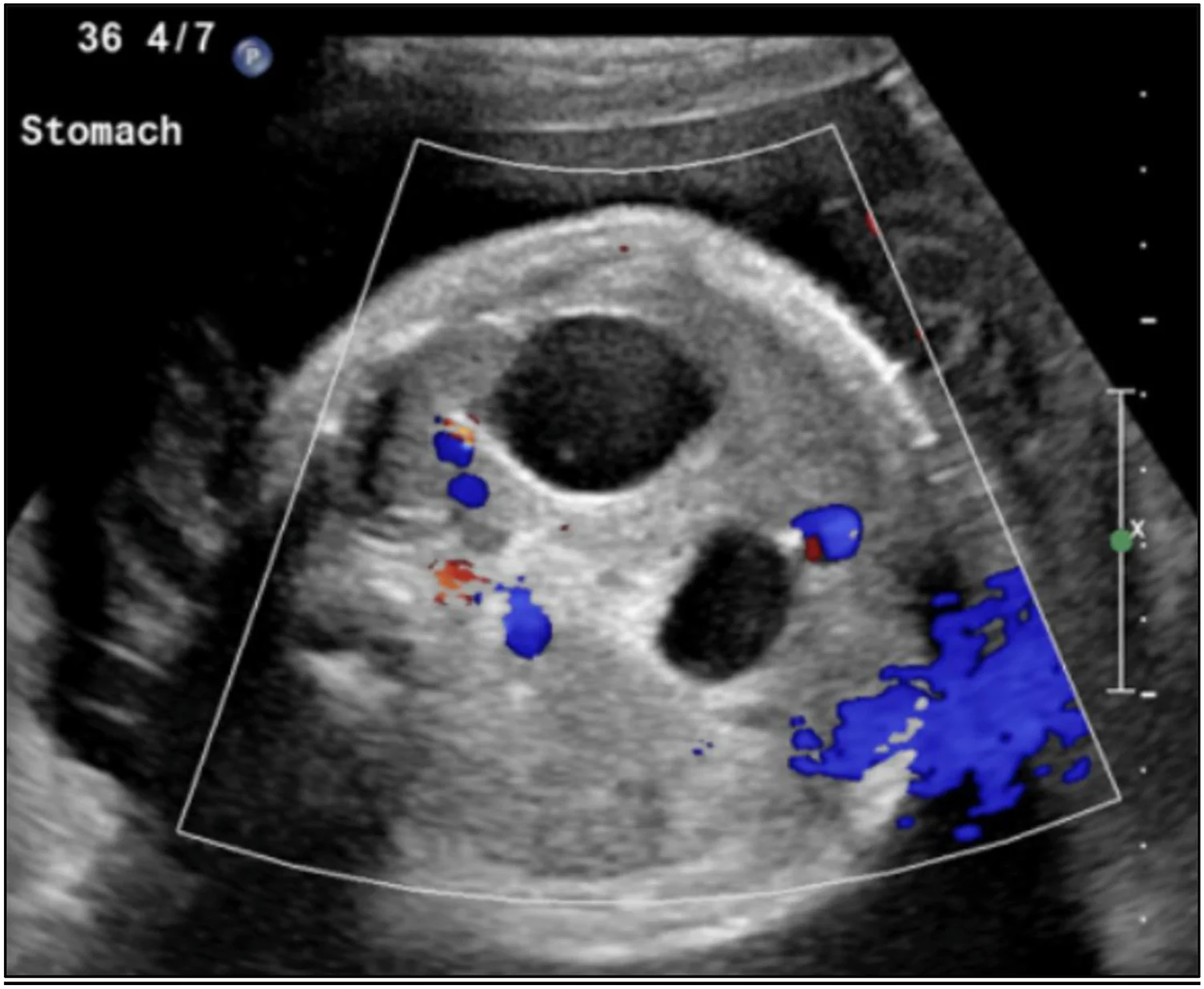
Fetal Ultrasound Showing Double Bubble Sign. Ultrasound at 36 weeks demonstrating a double bubble sign, concerning for duodenal atresia. Color doppler demonstrated no vascular flow within the lesion.

Given the severe polyhydramnios, the patient underwent induction of labor at 37 weeks 5 days at a tertiary care center. Her induction was initiated with a cervical balloon catheter and oxytocin infusion. She underwent artificial rupture of membranes, progressed to complete cervical dilation, and began pushing. Due to nonreassuring fetal heart tones, vacuum-assisted vaginal delivery was performed, resulting in a live-born male infant weighing 2800 grams. After birth, the infant was noted to have dysmorphic facial features consistent with Down syndrome. Duodenal atresia repair was completed on day of life 3 with no complications. Postnatal echo identified a moderate secundum atrial septal defect with bidirectional shunting.

Infant chromosomal analysis on peripheral blood with extended cell count (50 cells) confirmed the cytogenetic result of sporadic Down syndrome: 47,XY,+21 (Figure 2). No mosaicism was detected. The performing PGT-A laboratory was contacted at the time of postnatal diagnosis to clarify if any unreported mosaicism of the transferred embryo was detected; however, the laboratory stated that they do not report or disclose findings below their validated reporting threshold of 30%, as those results are not considered reliable for clinical interpretation. Written informed consent was obtained from the patient for publication of this case report and any accompanying images.

**Figure 2:**
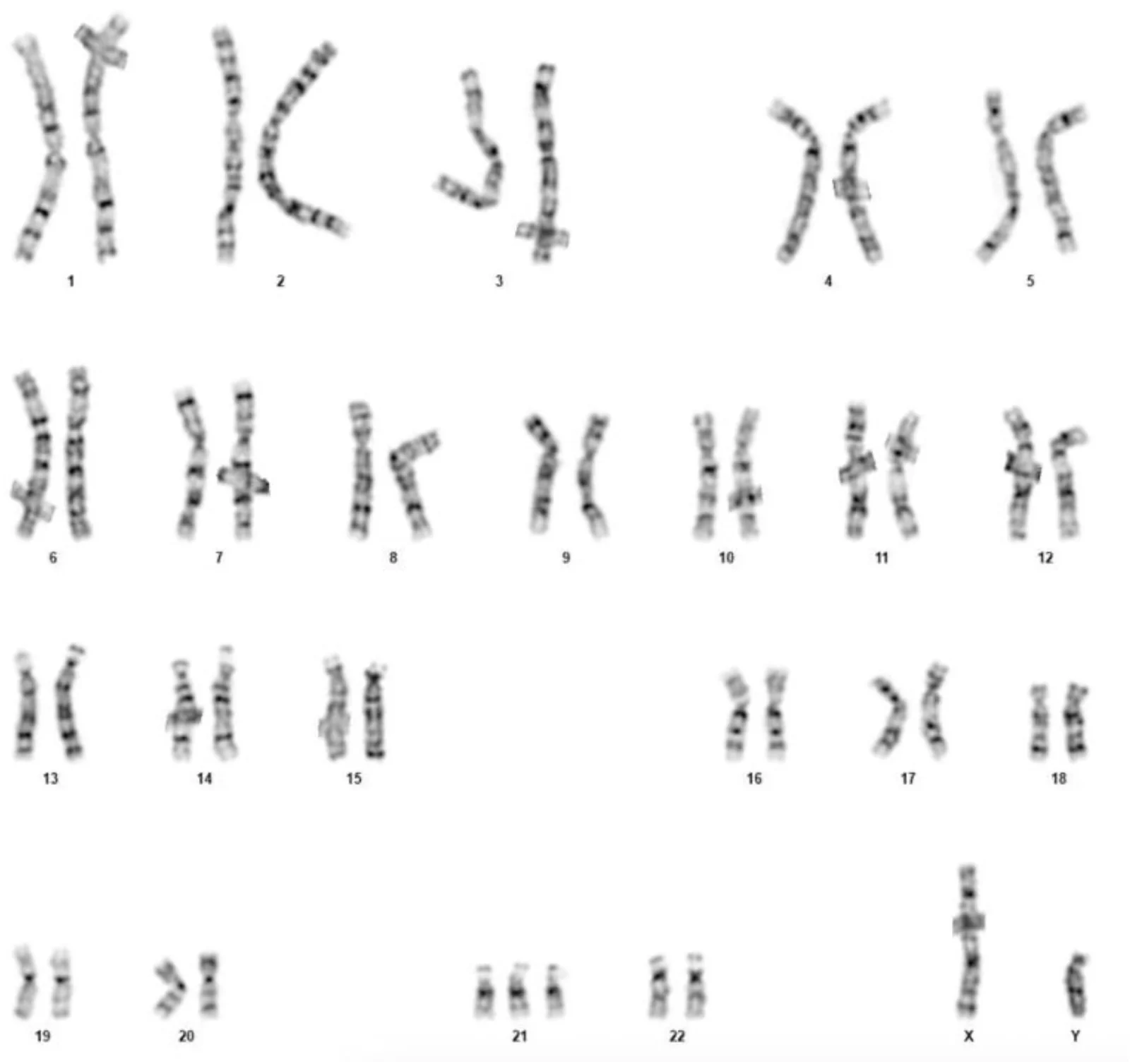
Postnatal Karyotype. Postnatal peripheral blood karyotype of the neonate demonstrating trisomy 21.

## Discussion

### Overview

This case represents a rare but clinically important scenario in which a patient received two independent, reassuring genetic screening results, yet delivered a chromosomally abnormal neonate. Each screening modality is individually highly accurate, with a negative predictive value (NPV) of 94% to 100% for PGT-A and both sensitivity and specificity exceeding 98.8% for trisomy 21 detection on NIPT (6, 7). The compounding false-negative results described in this case represent an unexpected manifestation of a known but rare limitation of these technologies.

### Limitations of PGT-A

PGT-A is performed via trophectoderm biopsy at the blastocyst stage, which consists of sampling 5 to 10 cells that are destined to become the placenta rather than the fetus itself (2). With next-generation sequencing (NGS), the process involves DNA amplification to determine chromosomal copy numbers (8). Mosaicism, defined as the coexistence of more than one cell lineage within the same embryo, represents one of the most significant sources of discordance between PGT-A and true fetal chromosomal makeup. Mosaic embryos exhibit chromosomal copy numbers that fall out of the normal disomic range but do not reach full monosomy or trisomy thresholds (2). The rate of mosaic results on trophectoderm biopsy varies widely from 2% to 20% depending on laboratory platform, DNA amplification method, analysis software, and reporting thresholds (8).

Interlaboratory variability further complicates test interpretation. Depending on the laboratory’s reporting threshold, an aneuploidy rate of 45% may be reported as euploid, low-level mosaic, or high-level mosaic (8). One study demonstrated that euploidy rates across independent laboratories performing PGT-A on identical blastocysts ranged from 52.3% to 73.6%, and mosaicism rates fluctuated more than fourfold (2). A recent systematic review and meta-analysis by Bacal et al. (6) reported an overall NPV of 94.2% for euploid embryos and a misdiagnosis rate of 0.2% after euploid embryo transfer. Although this rate is reassuring at the individual level, it is clinically meaningful given the volume of IVF with PGT-A performed worldwide each year. Sources of misdiagnosis include human error, DNA contamination, biopsy technique, mosaicism, and the rare possibility of spontaneous conception concurrent with embryo transfer (2).

A recent case series by Bai et al. (9) further illustrates these limitations, describing two couples who underwent IVF with PGT-A. Both patients had euploid embryo transfers followed by missed miscarriages, with products of conception demonstrating embryonic mosaicism. These cases reinforce that embryos classified as euploid may still harbor abnormal cell lines undetected by trophectoderm biopsy. Although PGT-A is a screening tool, it is often used to guide decisions with diagnostic consequences, including which embryos are transferred and which are discarded. Such consequences underscore the need for patients to understand the inherent limitations of these tests prior to initiation.

### Limitations of NIPT

NIPT analyzes cell-free fetal DNA in maternal plasma derived from apoptosis of the trophoblast layer of the placenta (7). Because cell-free DNA is released from the entire placenta, it is theoretically more representative of the true genetic makeup of the fetus compared with the small trophectoderm sample obtained by PGT-A (7). However, as NIPT also collects placental DNA, it is therefore subject to many of the same biological confounders as PGT-A. Confined placental mosaicism (CPM) is where chromosomally abnormal cells are restricted to the placenta while fetal chromosomal makeup is normal or euploid (10). CPM is the most widely recognized cause of false-positive NIPT and typically results in healthy pregnancies (10). The reverse situation is less commonly discussed but biologically plausible, where if the placenta is predominantly disomic for chromosome 21, whereas the fetus is trisomic, the NIPT may return with a falsely reassuring low-risk result.

Prior studies have shown that false-negative NIPT results are associated with low fetal fraction, typically <4% (10). In our patient, the fetal fraction of 10% was within the accepted adequate range for accurate interpretation, making low fetal fraction an unlikely explanation for the outcome. Madjunkov et al. (7) evaluated cfDNA screening after euploid embryo transfer and reported a low PPV of only 7.7%, where only one case of an abnormal NIPT resulted in a chromosomally abnormal fetus, while the remaining cases were concordant with the initial PGT-A results. This work supports the idea that euploid PGT-A reliably selects against aneuploidy in the majority of cases, but it does not preclude the possibility of a false-negative NIPT in the same pregnancy, as seen in our patient.

It is also important to note that NIPT is not designed to detect mosaicism. ASRM’s committee opinion on mosaic embryo management explicitly acknowledges the possibility of false-negative or false-positive NIPT results in mosaic pregnancies (8). The cfDNA detected by NIPT represents a snapshot of circulating placental DNA at the time of blood draw, and if the chromosomally abnormal cell line is underrepresented in the placenta relative to the fetus at that time, the abnormality may go undetected.

### Nonmosaic trisomy 21

The postnatal karyotype performed on peripheral blood for our patient confirmed sporadic trisomy 21 (47, XY,+21), with no mosaicism detected on extended cell count of 50 cells. This finding is particularly notable because it argues against a scenario in which low-level mosaicism was simply missed by both screening tests, though the possibility of mosaicism across other tissue types cannot be excluded as no additional sample types were evaluated. One plausible biological explanation is that the trisomy arose as a post-zygotic mitotic error early in embryonic development, resulting in embryonic mosaicism with subsequent lineage-specific segregation, such that the placenta remained predominantly euploid while the fetal lineage became trisomic (11). Alternatively, the embryo may have been initially trisomic, with subsequent trisomy rescue occurring in the placental but not the fetal lineage, yielding a euploid placenta and persistently trisomic fetus (11). Although unlikely, we also cannot preclude the possibility of technical or human error in the laboratory setting, such as the transfer of an unintended embryo.

This case has direct implications for how clinicians should counsel IVF patients before, during, and after genetic screening. Patients may develop a perception of confirmed genetic normalcy after receiving a euploid PGT-A result. Both ACOG and ASRM are explicit in that PGT-A is a screening tool, and ACOG recommends that all patients should be offered standard prenatal aneuploidy screening following PGT-A (3, 12). Counseling must include the option of invasive diagnostic testing, particularly if a screening test is abnormal. Invasive prenatal diagnosis via chorionic villus sampling or amniocentesis remains the only definitive method for confirming fetal karyotype; however, CVS samples placental tissue and thus may also be subject to CPM, which can result in discordance with the true fetal karyotype. Accordingly, shared decision-making regarding these options should be incorporated into the counseling of all IVF patients.

## Conclusion

This case illustrates a rare but clinically meaningful failure of two independent and highly accurate aneuploidy screening modalities in the same IVF-conceived pregnancy. PGT-A and NIPT are powerful tools for risk reduction, but they do not eliminate risk. Clinicians should counsel patients accordingly, offer diagnostic tests when indicated, and continue to report discordant cases in the literature to better characterize the true risk of aneuploidy after euploid embryo transfer.

## CRediT Authorship Contribution Statement

**Jennifer Law:** Writing – review & editing, Writing – original draft, Investigation, Data curation, Conceptualization. **Kyra Ramsey:** Writing – review & editing, Investigation, Data curation, Conceptualization. **Nicole Sprawka:** Writing – review & editing, Validation, Data curation. **Joana Perdigao:** Validation, Supervision, Data curation.

## Declaration of Interests

J.L. has nothing to disclose. K.R. has nothing to disclose. N.S. has nothing to disclose. J.P. has nothing to disclose.
